# Large-Scale Multi-Cancer Detection by Learning Segmentation from Reports

**DOI:** 10.21203/rs.3.rs-10131590/v1

**Published:** 2026-07-15

**Authors:** Pedro R. A. S. Bassi, Xinze Zhou, Wenxuan Li, Szymon Płotka, Jakob Wasserthal, Jieneng Chen, Ibrahim E. Hamamci, Sezgin Er, Jakub Prządo, Zheren Zhu, Gorkem Durak, Wiktoria Romańczyk, Yavuz B. Taktak, Melih Akan, Yuhan Wang, Scott Ye, Qi Chen, Ashwin Kumar, Lizhou Wu, Guang Zhang, Bjoern Menze, Jarosław B. Ćwikła, Yuyin Zhou, Frank H. Miller, Yunhe Gao, Akshay S. Chaudhari, Curtis P. Langlotz, Ulas Bagci, Sergio Decherchi, Andrea Cavalli, Arkadiusz Sitek, Kang Wang, Yang Yang, Alan L. Yuille, Zongwei Zhou

**Affiliations:** 1 Johns Hopkins University, Baltimore, MD, USA.; 2 Harvard Medical School, Boston, MA, USA.; 3 Massachusetts General Hospital, Boston, MA, USA.; 4 Jagiellonian University, Kraków, Poland.; 5 University Hospital Basel, Basel, Switzerland.; 6 University of Zurich, Zurich, Switzerland.; 7 ETH AI Center, Zurich, Switzerland.; 8 Istanbul Medipol University, Istanbul, Turkey.; 9 Warmian-Masurian Cancer Center, Olsztyn, Poland.; 10 University of California, Berkeley, CA, USA.; 11 University of California, San Francisco, CA, USA.; 12 Northwestern University, Illinois, USA.; 13 Medical University of Białystok, Poland.; 14 Istanbul University, Istanbul, Turkey.; 15 University of California, Santa Cruz, CA, USA.; 16 Stanford University, Stanford, CA, USA.; 17 Shandong Provincial Qianfoshan Hospital, Shandong, China.; 18 University of Warmia and Mazury, Olsztyn, Poland.; 19 Google, San Francisco, CA, USA.; 20 Istituto Italiano di Tecnologia, Genoa, Italy.; 21 University of Bologna, Bologna, Italy.; 22 École Polytechnique Fédérale de Lausanne, Lausanne, Switzerland.; 23 Johns Hopkins Medicine, Baltimore, MD, USA.

## Abstract

More than 300 million computed tomography (CT) scans are performed worldwide each year, yet many early or incidental tumors in these scans remain undetected. Artificial intelligence (AI) could help: segmentation models can surpass radiologists and alternative AI models in detecting tumors, and they localize the tumors for radiologist verification. However, segmentation-based tumor detection has long been limited by the need for tumor masks: radiologist-drawn tumor outlines that are scarce, expensive, and entirely unavailable for many cancer types. In contrast, nearly every CT scan is accompanied by a radiology report with detailed tumor descriptions. Yet, these reports have not been used effectively to train tumor segmentation models. Here, we introduce R-Super, a framework that converts routine radiology and pathology reports into localized training signals for tumor segmentation. R-Super trains AI to segment tumors that match their descriptions in reports. Reports are only needed for training, not inference. We trained R-Super on 127,496 CT-Report pairs (42 million 2D images, USA) and evaluated it internally at UCSF (N=2,301, USA) and externally at Stanford (N=1,976, USA), Medipol (N=1,327, Turkey), and Basel (N=2,935, Switzerland). R-Super detects 7 tumor types for which public tumor masks are scarce or absent: spleen, gallbladder, prostate, bladder, uterus, esophagus, and adrenal tumors. Training R-Super on over 100,000 reports (no mask) outperformed mask-based segmentation models trained on 870 masks, demonstrating that large-scale report-based training surpasses smaller-scale mask-based training. Alternatively, by training R-Super on both these reports and masks together, cancer detection sensitivity increased by over +11% beyond mask-only training, and DSC by +14%. R-Super significantly surpassed 6 alternative training frameworks trained on the same dataset and 9 leading public AI models. R-Super significantly surpassed six radiologists in detecting six tumor types and matched them for uterus tumors in a reader study. On average, R-Super detected 56% more malignant tumors than the radiologists, at matched false positive rate. These results show that radiology reports are not merely clinical documentation, but a large-scale, underutilized source of localized supervision for training more accurate cancer detection AI. By effectively learning from reports, R-Super enables tumor segmentation models to scale beyond scarce radiologist-drawn tumor masks and advances automated and incidental cancer detection closer to clinical deployment. We release code, over 22,000 CT scans and reports, and the first public AI model to reach or surpass radiologist performance in detecting these tumor types on CT.

## 1 Main

Cancer is a leading cause of death worldwide [1, 2], and early detection is crucial: five-year survival often exceeds 90% when tumors are detected early but drops below 20% once they become advanced or metastatic [3]. Each year, over 300 million Computed Tomography (CT) scans are performed globally [4], to evaluate suspected cancer and for many other reasons. In both cases, this enormous imaging volume registers numerous early-stage tumors that often go undetected, even by expert radiologists. For instance, in a study of CT scans taken before pancreatic cancer diagnosis, about 50% of the tumors were missed by radiologists [5]. Artificial Intelligence (AI) can assist radiologists in incidental or early tumor detection [6–8]: AI can perceive subtle tumor signs imperceptible to humans, analyze images in 3D rather than slice by slice, and has been shown to surpass radiologists in detecting pancreas [7, 9] and stomach [8] tumors on CT.

**Fig. 1:**
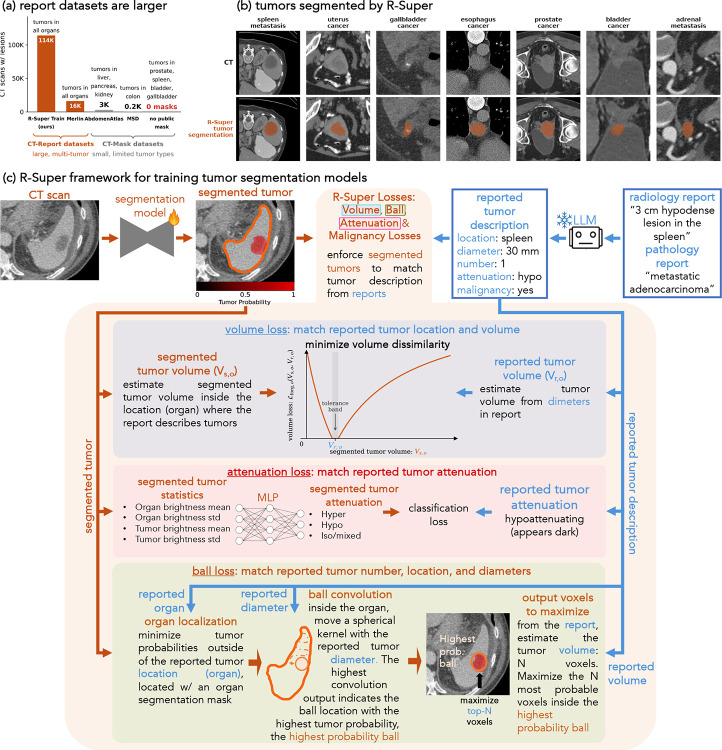
**(a) R-Super learns from reports because reports are more abundant than tumor masks**. Our training set has 127K CT-Report pairs, 114K with tumors; Merlin [22] has 25K, 16K with tumors. The largest public CT-Mask datasets have up to 3K CT-Mask pairs with tumors. Many tumor types have no public masks. **(b) Examples of tumors segmented by R-Super** on CT scans from the external Medipol test set (Tab. 1). **(c) Overview of R-Super training framework**. First, an LLM (frozen) extracts tumor characteristics from radiology and pathology reports. Then, four new loss functions teach a tumor segmentation model to segment tumors that match these tumor characteristics. The **Volume Loss** (Sec. 4.3) enforces the volume of segmented tumors to match the report. From the tumor diameters in the radiology report, we estimate the total reported tumor volume in each organ $o,V_{r,o}$. We then sum the segmentation model output over all voxels inside organ o (located with a pre-saved AI-made organ mask) to estimate the segmented tumor volume, $V_{s,o}$. Finally, we encourage $V_{s,o}$ to match $V_{r,o}$ by minimizing a custom dissimilarity function (Eq. 8). The **Ball Loss** (Sec. 4.4) encourages segmented tumors to match the tumor number, rough locations, and diameters from the report. Each reported tumor is located by a Ball Convolution. It slides a fixed spherical kernel (a ball with the reported tumor diameter) over the segmentation output, summing the predicted tumor probabilities inside each sphere position. The convolution maximum is the most likely location for the reported tumor. Within this location, we maximize the top-N most probable voxels, where N is the tumor volume from report. These top-N voxels can have any shape within the reported tumor diameter. The Ball Loss locates and optimizes each tumor documented in the report, avoiding overlaps. The **Attenuation Loss** (Sec. 4.5) enforces segmented tumors to match the tumor attenuation (relative brightness) from the report. The **Malignancy Loss** (Sec. 4.6) distinguishes benign from malignant tumors using supervision from pathology reports if available.

State-of-the-art AI for tumor detection is typically formulated as semantic segmentation [7–11], in which tumors are detected and localized by outlining them directly on the medical image. Compared with classification models, segmentation models are generally more accurate and more robust across hospitals [12, 13]. Their output is also clinically interpretable, because tumor outlines allow radiologists to inspect and verify the AI decision. However, training segmentation models requires *tumor masks*: voxel-wise tumor outlines drawn by radiologists. Creating these masks is slow, costly, and not part of standard radiologist work. Manually outlining a tumor on a single 3D CT scan can take over 30 minutes. In Xia *et al*. [6], generating 3,125 pancreatic tumor masks required eight radiologists, five years, and millions of dollars. Semi-automated and interactive contouring tools can reduce this burden, but they still require labor-intensive radiologist supervision and annotations [14]. As a result, public CT datasets contain few tumor masks (Fig. 1), which are concentrated in few organs, such as the kidney, liver, lungs, and pancreas [15–18]. For many other organs, including the spleen, gallbladder, prostate, and bladder, no public tumor masks exist. For the uterus, esophagus, and adrenal glands, fewer than 60 public tumor masks exist [19, 20]. Consequently, public AI models are not accurate in detecting these seven tumor types, performing substantially below radiologists (Tab. D20, D48).

While tumor masks are scarce, nearly every CT scan is already accompanied by a radiology report. These reports routinely describe tumor number, size, attenuation, and approximate anatomical location, while pathology reports provide confirmation of malignancy. CT-Report pairs are naturally orders of magnitude more abundant than CT-Mask pairs: public datasets already contain over 24,000 CT-Report pairs [21, 22], whereas the largest public CT-Mask datasets rarely exceed 1,000 tumor masks [15–18]. Similarly, hospitals may contain millions of CT-Report pairs, but few or no tumor masks. Reports describe tumors across all organs, while public tumor masks are limited to a few organs. This motivates a central question: *Can reports supplement, or even substitute, tumor masks in training AI for tumor segmentation?*

Reports have been used to train AI models for cancer-related tasks. Reports are commonly used to train foundation vision-language models (VLMs), including Google’s MedGemma [23], Alibaba’s Lingshu [24], Microsoft’s MedImageInsight [25], and Stanford’s Merlin [22]. Alternatively, labels extracted from reports are often used to train classifiers. However, VLMs and classifiers treat reports primarily as non-localized supervision: training signals that directly inform the AI model whether a tumor is present, but not where it is localized within the CT scan space. As a result, classifiers and VLMs can detect or describe tumors, but cannot provide per-voxel tumor localization. This limits a radiologist’s ability to verify these AI models’ decisions. Furthermore, classifiers and VLMs often miss substantially more tumors than segmentation models [10, 12]. This suggests that localized supervision is important for accurate tumor detection: tumor masks inform segmentation models exactly where tumors are, helping them learn to detect tumors that may occupy less than 0.0001% of a 3D CT scan. This raises the question of whether, like tumor masks, radiology reports can provide the localized supervision.

Here, we propose R-Super (Report Supervision), a training framework that converts radiology and pathology reports into localized supervision to train tumor segmentation models on large-scale CT-Report datasets (Fig. 1). The challenge is that, unlike tumor masks, reports do not provide voxel-wise tumor locations. However, reports provide detailed descriptions of tumors, including tumor number, rough location, size, attenuation, and malignancy. R-Super transforms these descriptions into localized training signals through new loss functions, which teach segmentation models to segment tumors consistent with their report descriptions. Therefore, R-Super can learn tumor segmentation from reports only, without masks. When some masks are also available, R-Super can train with both CT-Report and CT-Mask pairs. Reports are used only during training, not inference, and the R-Super training framework can be applied to any segmentation architecture. Thus, R-Super directly addresses the main bottleneck in tumor segmentation: the scarcity of tumor masks.

To train R-Super, we created a new dataset, comprising 127,496 CT-Report pairs (42 million 2D slices), primarily extracted from 27 years of archived data from the UCSF hospital and its affiliated institutions in the USA. We evaluated R-Super across four hospital cohorts in three countries: UCSF (N=2,301, USA), Stanford (N=1,976, USA), Medipol University Hospital (N=1,327, Turkey), and University Hospital Basel (N=2,935, Switzerland). We studied seven tumor types with few or no public CT tumor masks: spleen, gallbladder, prostate, bladder, uterus, esophagus, and adrenal tumors. *Even without tumor masks, R-Super substantially outperforms 9 leading public AI models in tumor detection and segmentation*, surpassing public VLMs by Google, Stanford, Alibaba, and Microsoft by at least +18% balanced accuracy in malignant tumor detection in external validation, and surpassing public segmentation models (ULS and FLARE) by at least +9% balanced accuracy and +31% DSC (Sec. 2.1). R-*Super shows reports can substitute or complement tumor masks*: training on over 100,000 CT-Report pairs surpassed training on 870 CT-Mask pairs (created by 31 radiologists). Training on these reports and masks together surpassed mask-only training by at least +11% malignant tumor detection sensitivity and +14% segmentation DSC (Sec. 2.2; Supp. D.2). *R-Super is more effective in learning from reports*: it surpassed 6 alternative training strategies also trained on our dataset by +5% to +12% detection sensitivity and +14% segmentation DSC (e.g., report-based CLIP, multi-task learning, and classification). *R-Super surpasses radiologist performance*: in a reader study, R-Super outperformed six radiologists in detecting six of seven tumor types, and matched them for uterus tumors. It detected 56% more malignant tumors than the radiologists on average, at matched specificity (Fig. 5). These results suggest that R-Super could help radiologists detect tumors that would otherwise be missed. R-Super also surpassed radiologist performances in the literature (Supp. B). To the best of our knowledge, R-Super is the first public AI model shown to surpass (or even reach) radiologist performance for detecting these tumor types on CT.

The central insight of this study is that medical reports are not merely clinical documentation or image-level labels, but an abundant source of localized supervision. Across other AI fields, such as NLP and computer vision, major advances like ChatGPT and CLIP came from shifting from small manually annotated datasets to massive datasets supervised by readily available text or captions [26, 27]. Our results (Fig. 3a) suggest that a similar transition is possible in cancer detection and segmentation: from training on hundreds of expensive CT-Mask pairs to training on hundreds of thousands of CT-Report pairs readily available in hospitals. By transforming reports into localized supervision, R-Super enables tumor segmentation models to scale beyond the longstanding limitation of scarce tumor masks. More broadly, R-Super offers a more interpretable and accurate alternative to classification and current VLMs for multi-tumor detection, enabling public AI models to surpass radiologist performance. This paradigm can advance opportunistic cancer detection by flagging probable cancer cases that would otherwise remain undetected on routine CT, enabling timely follow-up examinations. Together with the release of code, trained models, over 22,000 CT-Report pairs (chest, abdomen, and pelvis), and tumor masks for multiple tumor types unavailable in public segmentation datasets, this work provides an open-source foundation for broader automated and incidental cancer detection.

## 2 Results

We evaluated R-Super on four tasks: tumor detection, that is, assessing whether each organ contains tumors (benign + malignant tumor vs no-tumor); malignant tumor detection, that is, tumor detection stratified for malignant tumors (malignant vs no-tumor); malignancy identification, that is, detecting tumors and identifying whether they are malignant (malignant vs benign + no-tumor); and tumor localization, that is, determining whether detected tumors overlap with tumor masks drawn by radiologists.

Malignancy is confirmed with pathology reports in the UCSF test set. In the Stanford, Medipol, and Basel test sets, malignancy is confirmed from radiology reports, considering lesions described as malignant with high certainty or already diagnosed active cancer documented in the patient’s clinical history. In the reader study test set, malignancy is confirmed using either pathology reports or documentation of already diagnosed active cancer in radiology reports (dataset details in Sec. 4.1).

R-Super produces tumor detection and malignancy identification outputs using a classification head applied to the tumor segmentation output (Supp. C.1). Tumor detection through segmentation improves accuracy (Fig. 3) and interpretability. For alternative segmentation models without classification heads (ULS, FLARE, and segmentation baselines), we computed tumor detection metrics by thresholding the segmentation output, following prior work [10].

Section 2.1 compares R-Super trained on reports only (no mask) to 9 public AI models in tumor detection. Section 2.2 shows how R-Super performance can be further scaled by training with CT-Mask and CT-Report pairs, and evaluates tumor localization. Section 2.3 compares R-Super to 6 alternative training methods also trained on our dataset. Finally, Sec. 2.4 compares R-Super to radiologists in a reader study. This paper includes three tasks, four test datasets, and comparisons to 15 alternative models. Given the large number of experiments, this Results section presents the main findings, while detailed results tables, additional analyses, and ablation studies are in Supp. D. Ablations show that all the R-Super loss functions positively contribute to the model performance (Supp. D.5). R-Super can train any segmentation architecture. Here, we chose MedFormer [28], for its success in previous benchmarks (Touchstone [29]). Data pre-processing and training details are in Supp. E.

### 2.1 Detect 7 tumor types without any tumor masks

**Fig. 2:**
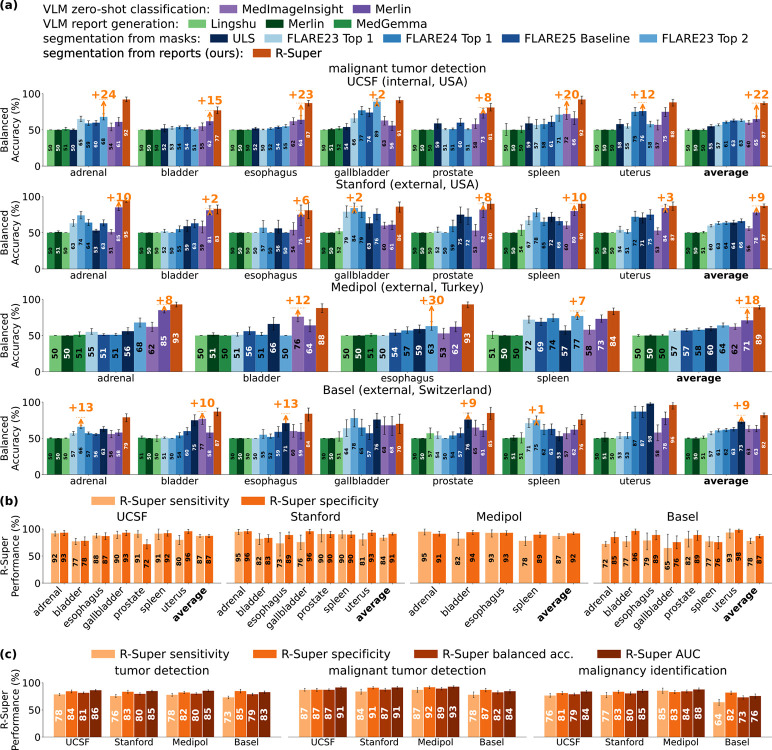
**(a) R-Super, trained with reports only (no mask), surpasses leading public AI models in multi-tumor detection, by large margins**. R-Super surpassed the public AI models by +9% to +22% average balanced accuracy. In this figure, we evaluate the AI models for malignant tumor detection (malignant vs no-tumor). We skipped prostate, uterus, and gallbladder tumors in the Medipol test set, where the number of these malignant tumors was insufficient (Tab. A1). The plot shows balanced accuracy, at the best operating point for each model. The results of all models represent external evaluation on a hospital not seen in training, except for R-Super evaluated at UCSF, and Merlin evaluated at Stanford. Details on the VLM evaluation procedure are available in Supp. F. **(b) Sensitivity and specificity of R-Super** in malignant tumor detection (malignant vs no-tumor). **(c) Performance of R-Super in tumor detection, malignant tumor detection, and malignancy identification**. Detailed results for all models are available in Supp. D.6. Dataset details are in Tab. 1 and Tab. A1. Error bars represent 95% confidence intervals, calculated with bootstrapping (1,000 bootstrap samples).

We evaluated R-Super in the detection of 7 tumor types: spleen, gallbladder, prostate, bladder, uterus, esophagus, and adrenal gland tumors, 7 important tumor types underrepresented in public segmentation datasets. We compared R-Super with 9 leading public AI models, showing that learning tumor segmentation from reports allows R-Super to set a new state-of-the-art for open-source AI in tumor detection. Results are summarized in Fig. 2. Here, we trained R-Super on only CT-Report pairs (no mask). Medical AI often loses performance when tested on hospitals not seen during training, making evaluation on external (unseen) hospitals critical [6, 13]. Therefore, we evaluated R-Super on 3 external hospitals: Stanford Test (N=1,976, USA), Medipol (N=1,327, Turkey), and Basel (N=2,935, Switzerland); plus one internal: UCSF Test (N=2,301, USA). For the evaluation on Stanford Test, we trained R-Super on the UCSF Train set (N=116,514, USA). For evaluation on UCSF Test, Medipol, and Basel, we trained on the full R-Super train set (N=127,496, combining UCSF and Stanford data). Stanford data derives from the Merlin dataset [22], the largest public abdominal CT dataset, and, up to now, the only public dataset with an adequate number of the tumor types evaluated here. Details on all datasets are in Tab. 1 and A1. The Medipol and Basel test sets provide a particularly challenging test of generalization: these datasets include only images from Asia and Europe, but R-Super was trained only on North American images.

**R-Super substantially surpassed 9 public AI models in tumor detection** (Fig. 2 **and Supp. D.6), including leading medical VLMs**, namely Alibaba’s Lingshu [24], Google’s MedGemma [23], Microsoft’s MedImageInsight [25], and Stanford’s Merlin [22]. In malignant tumor detection (malignant vs no-tumor), R-Super significantly (p<0.05) surpassed the best public AI model by +9% to +22% average balanced accuracy across all the four test sets (Fig. 2). Specifically in Medipol and Basel, R-Super outperformed all VLMs by +18% to +19% average balanced accuracy. These two datasets are important because they represent external validation for both R-Super and all VLMs (Merlin was trained at Stanford). In tumor detection (malignant + benign vs no-tumor), R-Super significantly (p<0.05) surpassed the best public AI model by +10% to +19% average balanced accuracy (Supp. D.6). Among the public VLMs, zero-shot classification [30] performed substantially better than report generation. For instance, when evaluated with zero-shot classification, Merlin was the strongest public model (after R-Super) in 3 test sets. However, Merlin and the other VLMs missed almost all tumors when evaluated through report generation, with average sensitivity below 10% in every dataset (Supp. D.6). In contrast, R-Super achieved high sensitivity and specificity in all datasets. In malignant tumor detection, it achieved sensitivity and specificity of 87% (95% CI of 84–90%) and 87% (84–90%) in the UCSF test set, 84% (79–88%) and 91% (89–93%) in Stanford, 87% (82–91%) and 92% (89–94%) in Medipol, and 78% (73–83%) and 87 (83–90%) in Basel (detailed results in Fig. 2 and Supp. D.1). R-Super could even match or surpass human experts in a reader study (Sec. 2.4).

#### R-Super also surpassed public segmentation models by large margins.

Our 9 baselines include multiple leading public segmentation models: the Universal Lesion Segmentation model (ULS) [31], the top two segmentation models in the FLARE 23 challenge [32], the top FLARE 24 model [33], and the FLARE 25 baseline model [34]. Unlike R-Super, these models were trained only with tumor masks, not reports, and their training datasets do not distinguish tumor type or malignancy. These public segmentation models underperformed in our study (Fig. 2), suggesting that the tumor types evaluated here are underrepresented in the FLARE and ULS datasets. This finding highlights how limited tumor mask availability remains a key bottleneck for open research on multi-tumor detection and localization. As an additional limitation, ULS was only trained on images cropped around tumors [31], missing no-tumor controls, which can limit tumor detection performance. Even so, ULS performed comparably to the FLARE models (Fig. 2). Overall, our results (Fig. 2, Supp. D.6) indicate that R-Super is, to our knowledge, the first public AI model capable of reliably detecting these 7 tumor types on CT.

#### Besides detecting tumors, R-Super identifies whether they are malignant.

In malignancy identification (malignant vs. benign + no-tumor), R-Super achieved on average: 84% (81–86%) AUC on UCSF test (internal, USA), 85% (82–88%) on Stanford test (external, USA), 76% (71–79%) on Basel test (external, Switzerland), and 88% (85–90%) on Medipol test (external, Turkey). Results are summarized in Fig. 2 and detailed in Tab. D6. The public AI models were not evaluated for malignancy identification, because the public segmentation models were not trained for this task, and public VLMs had insufficient tumor detection performance (tumors must first be detected before malignancy can be assessed). Malignancy identification is a very challenging task even for humans, and the average performance of R-Super surpassed radiologists in this task (Sec. 2.4). However, AI-based tumor detection is arguably more critical than malignancy identification: a detected tumor of unknown malignancy can be characterized as malignant or not through follow-up exams, but an undetected tumor may go untreated.

### 2.2 Scale mask datasets

R-Super allows training a tumor segmentation model directly from radiology reports, without tumor masks. In turn, this trained segmentation model can help radiologists to create tumor masks faster: R-Super creates masks, and radiologists (with access to radiology and pathology reports) verify and correct these AI-made masks. R-Super is then fine-tuned on these verified CT-Mask pairs, plus all CT-Report pairs, further improving performance and accelerating annotation. This forms a report-based active learning loop (human-in-the-loop strategy). Traditional active learning has a cold-start problem: it needs the manual creation of many masks, from scratch, before the segmentation model becomes accurate enough to help radiologists. R-Super avoids this by first training on reports alone, then progressively improving as more masks become available. Additionally, we proposed comparing AI-made masks and reports to identify potential AI errors and prioritize these cases for correction. Our report-based active learning strategy is explained in Supp. C.2.

With R-Super-based active learning, 31 radiologists created 1,420 masks for the R-Super Train dataset and 870 masks for UCSF Train (details in Tab. 1). With the support of R-Super, tumor mask creation was 5 times faster for small tumors and 3 times faster for large tumors (Supp. C.2). To the best of our knowledge, our dataset provides the first public CT tumor masks for bladder, gallbladder, spleen, and prostate tumors. For uterus, esophagus, and adrenal tumors, existing public CT datasets contain at most 53 masks per tumor type [19, 20], whereas our training dataset provides from 176 to 288 masks for each of these tumor types (Tab. 1).

#### Even when CT-Mask pairs are available, CT-Report pairs substantially improve tumor detection.

**Fig. 3:**
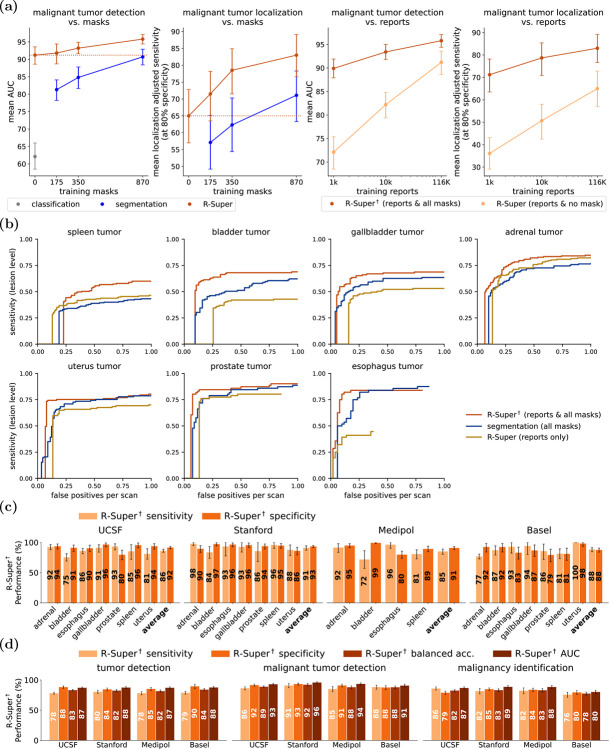
Training with many reports surpasses training with few masks; CT-Report plus CT-Mask training surpasses CT-Mask training. **(a) Scaling: R-Super performance improves with more training reports or masks**. R-Super trained on reports alone (116,514) surpassed a segmentation model trained on 870 masks for tumor detection, and one trained on 350 masks for tumor localization. For any number of training masks, adding CT-Report pairs to training substantially improved performance. Two left plots: scaling training masks. Two right plots: scaling reports. localization adjusted sensitivity: percentage of scans where AI-generated masks overlap radiologist-drawn masks. The segmentation model (blue) was trained on CT-Mask pairs only. Models were trained on UCSF train, and externally validated on Stanford test (N=1,976 scans; N=602 masks used for localization evaluation; Tab. 1). **(b) R-Super trained on reports and masks achieves higher FROC (tumor-level localization) than mask-only segmentation training for all tumor types**. For adrenal and uterus tumors, R-Super surpasses mask-only even if trained on reports alone. DSC in Fig. D6 and D5. **(c) Sensitivity & specificity of R-Super**^**†**^ (trained with CT-Report and CT-Mask pairs) in malignant tumor detection (malignant vs no-tumor). **(d) Performance of R-Super**^**†**^ **in tumor detection, malignant tumor detection, and malignancy identification**. Detailed results in Supp. D.1. Error bars represent 95% confidence intervals, calculated with bootstrapping (1,000 bootstrap samples).

Training R-Super with CT-Report and CT-Mask pairs provides substantially better performance than training with just CT-Mask pairs. We trained R-Super with all CT-Report pairs in UCSF train (116,514), plus different numbers of CT-Mask pairs (0, 175, 350, and 870). We compared it to a standard segmentation model trained with only these CT-Mask pairs (no report), using the same training parameters and AI architecture as R-Super (MedFormer). Figure 3a (detailed results in Fig. D4) shows that R-Super’s malignant tumor detection AUC significantly (p<0.05) and substantially surpassed the segmentation model both when few masks and when many masks were available in training. Furthermore, the R-Super model trained with all available reports and masks, named R-Super^†^, substantially surpassed the segmentation model trained with all masks (no reports) on all test sets (UCSF, Stanford, Medipol, and Basel), in tumor detection, malignant tumor detection, and small tumor detection (Supp. D.7). For instance, in malignant tumor detection, R-Super^†^ surpassed the segmentation model by +11% to +13% average sensitivity across the 4 test sets, with both models evaluated at 90% specificity (Tab. D32 to D35). The performance of R-Super^†^ is shown in Fig. 3c and 3d, and detailed in Supp. D.7. In malignant tumor detection, R-Super^†^ achieved from 86% to 91% sensitivity and 88 to 92% specificity (Fig. 3c).

#### CT-Report pairs substantially improve tumor localization.

R-Super localizes tumors by segmenting them. Tumor localization allows radiologists to visualize and verify the AI’s predictions. We evaluated tumor localization by comparing R-Super^†^’s tumor segmentation outputs to 602 tumor masks drawn by our collaborating radiologists on the external Stanford test set (Tab. 1). R-Super^†^, trained on both masks and reports (UCSF Train), significantly outperformed a segmentation model trained on masks alone in tumor localization: at 175, 350, and 870 training masks, R-Super^†^ achieved +14%, +16%, and +12% higher localization adjusted sensitivity (Figure 3 and Fig. D5) and +8%, +15%, and +14% higher DSC (Fig. D6) in Stanford Test. localization adjusted sensitivity measures the percentage of scans where the AI tumor segmentation output overlaps with radiologist-made tumor masks (evaluated at 80% specificity in Fig. 3). DSC quantifies the amount of overlap between the AI tumor segmentation output and the radiologist-made tumor masks. Overall, results show that including reports in the training dataset substantially improves tumor localization and segmentation.

#### Training on many reports surpasses training on fewer masks.

In tumor detection, R-Super trained on 116,514 reports and no mask surpassed the segmentation model trained on 175, 350, and 870 masks and no report (Fig. 3, external Stanford Test set). In tumor localization, R-Super (no mask) surpassed the segmentation model trained on 175 and 350 masks, in both localization adjusted sensitivity (Fig. 3) and DSC (Fig. D6). Therefore, large-scale report supervision can surpass smaller-scale mask supervision. Importantly, dataset size is not enough for a report-based model to surpass a mask-based model, training strategy is also critical. For instance, a classification model trained on 116,514 reports performed below a segmentation model trained on 175 masks in tumor detection, and much below R-Super (Fig. 3). This classification model was trained on the same dataset as R-Super, using classification labels extracted from all reports (tumor vs no-tumor labels, perorgan), and its architecture was similar to R-Super (MedFormer encoder). Its lower performance shows that a training framework that effectively learns from reports is essential for scaling tumor detection performance. With R-Super, training on reports alone can substitute for training on hundreds of radiologist-drawn masks. When both are available, combining CT-Report and CT-Mask pairs is optimal (R-Super^†^).

#### R-Super performance scales with reports.

We trained R-Super models with 1,000, 10,000, and 116,514 CT-Report pairs from UCSF Train. More CT-Report pairs in training improved the tumor detection and localization performance, as shown in Fig. 3 (Stanford Test set). This performance improvement was seen both when training R-Super with CT-Report pairs alone and when training with CT-Report plus CT-Mask pairs. The performance improvement was not saturated in the Fig. 3, even at 116,514 training CT-Report pairs. This result suggests that R-Super can continue improving as larger multi-hospital CT-Report datasets become available.

### 2.3 Surpass alternative training methods

We compared R-Super to 6 alternative training methods trained on the datasets we created. CLIP: contrastive pretraining on CT-Report pairs followed by CT-Mask fine-tuning for segmentation [30]; multi-task learning (MTL): joint segmentation and classification training from CT-Mask and CT-Report pairs [35]; Models Genesis: self-supervised CT-only pretraining followed by CT-Mask fine-tuning for segmentation [36]; nnU-Net: standard segmentation training from CT-Mask pairs [37, 38]; segmentation: standard segmentation with MedFormer architecture [28]; and classification: classification from report-derived labels (tumor vs no-tumor, per-organ).

Many of these alternative training methods can also learn from CT-Report pairs. However, they do not use reports to directly supervise segmentation, unlike R-Super. All alternative training methods used the same training datasets and segmentation architecture as R-Super (MedFormer [28]), and similar training procedures, except for nnU-Net, which used its automatically defined training parameters and the ResEncL architecture [38]. Regarding architecture modifications, the classifier was based on MedFormer’s encoder, MTL attached a classification head to the MedFormer’s output features, and all other training methods trained MedFormer without modifications. Each method was trained with all available data for its supervision type: CLIP and MTL used CT-Report and CT-Mask pairs; Models Genesis used all CT scans and CT-Mask pairs; report-based classification used CT-Report pairs; and standard segmentation and nnU-Net used CT-Mask pairs.

**Fig. 4:**
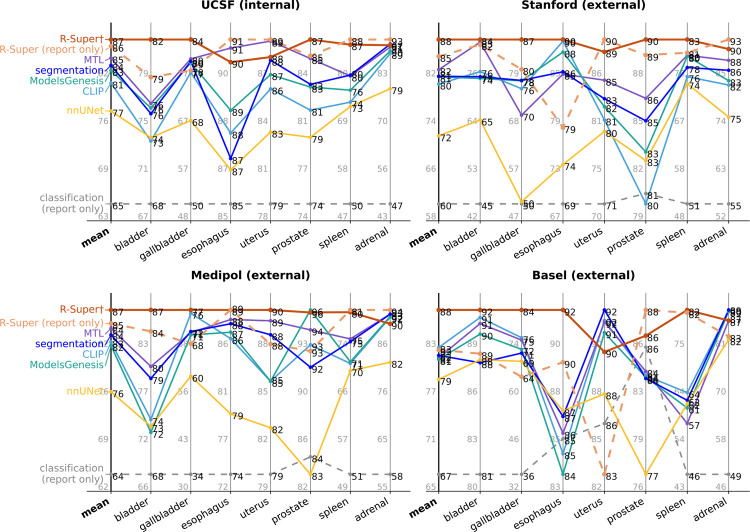
By effectively learning from reports, R-Super surpasses alternative methods trained on the same dataset. The plot compares the tumor detection AUC of R-Super to six alternative training methods. R-Super surpassed all alternative methods on average, on all datasets. All methods were trained on the same data as R-Super, using all available data for its supervision type: we include methods that learn from reports only (R-Super and classification), reports & masks (R-Super^†^, CLIP, and MTL), masks only (segmentation and nnU-Net), and unlabeled images (Models Genesis pre-trains with CT only, fine-tunes with CT-Mask pairs). We trained on UCSF Train for validation in Stanford, and on the R-Super Train dataset for validation on the other 3 test sets. Dataset details are in Tab. 1 and A1.

R-Super^†^ surpassed all alternative training methods in tumor detection, achieving significantly higher (p<0.05) average AUC in all test sets (Fig. 4, Supp. D.7). Against the second-best training method (MTL), R-Super^†^ improved DSC by +14% (Supp. D.2) on Stanford, and improved tumor detection sensitivity by +12% on UCSF (internal, Tab. D28), +5% on Stanford (external, Tab. D29), +6% on Medipol (external, Tab. D30), and +10% on Basel (external, Tab. D31), at matched 90% specificity. In malignant tumor detection, R-Super also surpassed all alternative training methods in all datasets (Supp. D.7). By using reports to directly supervise segmentation, R-Super’s tumor detection performance surpasses training methods that use reports to supervise other tasks (e.g., classification or CLIP).

### 2.4 Surpass radiologists and detect small tumors

To show that AI has potential to assist radiologists, we first need to demonstrate that it can detect tumors they miss. To this end, Supp. B shows that R-Super^†^ substantially surpassed radiologist performance published in the literature for detecting at least 5 tumor types (adrenal, bladder, esophagus, gallbladder, and prostate) and on average. However, comparisons to the literature are limited, since we cannot evaluate R-Super^†^ on the same datasets used to evaluate radiologists in the literature (private datasets).

Therefore, we conducted a reader study, where we evaluated R-Super^†^ and 6 radiologists on the same dataset. It contains 637 CT scans from 513 patients, 50% from UCSF (USA), 30% from Stanford (USA), and 20% from Medipol (Turkey); 132 scans have no tumor, and 505 have tumors, of which 47% include only small tumors (≤ 2 cm in diameter, Tab. A1). This large proportion of small tumors makes this dataset challenging by focusing on early cancer detection. Additionally, this reader study includes 50% of external data, from Stanford and Medipol. When externally evaluating on Stanford and Medipol data, we used R-Super^†^ trained on UCSF Train. When internally evaluating on UCSF data, we used R-Super^†^ trained on R-Super Train. Dataset details in Tab. 1. All cancer patients had mentions of known (already diagnosed) active cancer in their radiology reports or clinical history, or had positive pathology reports. Because the reader study dataset is large, each radiologist independently read half of the dataset (randomly selected). As a result, each CT scan was read by three radiologists, and radiologist performance is averaged across these three reads. The results for each individual read are in Supp. D.8. The readers are six radiologists with 2 to 8 years of experience (Tab. D40).

For each scan, radiologists answered two questions: (Q1) which organs contained a tumor, generically defined as any focal lesion, and (Q2) which organs contained a malignant tumor. The reader study instructions are in Supp. H. We used Q1 to evaluate tumor detection and malignant tumor detection. For tumor detection (benign + malignant vs no-tumor), radiologists were correct if they detected any tumor in a scan with a benign or malignant tumor. For malignant tumor detection (malignant vs no-tumor), radiologists were correct if they detected a tumor in a scan with a malignant tumor, regardless of whether they called it malignant; scans with only benign tumors were excluded. We used Q2 to evaluate malignancy identification (malignant vs benign + no-tumor): radiologists were correct if they called a malignant tumor malignant, and did not call any tumor malignant in scans with no tumor or only benign tumors.

In Q1, radiologists marked detected tumors as probable or certain. For the probable level, radiologists were instructed to include any tumor they would mention in a radiology report, simulating which tumors would be detected in clinical practice. In Q2, they also marked malignancy as probable or certain. In this section, we focus on the probable level, considering a tumor (or malignancy) detected if the radiologist marked it as probable. Our primary goal is to test whether AI can reduce completely missed tumors. Once detected, the existence and malignancy of uncertain tumors can be further confirmed through follow-up imaging or other examinations. Detailed results at the certain level are in Supp. D.8.1. At the certain level, radiologists achieved very high average specificity in malignant tumor detection (99%, 95% CI: 98–99%) but low sensitivity (41%, 37–45%). At the same 99% specificity, R-Super^†^ significantly (p<0.05) increased sensitivity by +25% on average, achieving sensitivity of 66% (CI: 59–72%, Tab. D50).

#### R-Super^†^ detected 56% more malignant tumors while maintaining the same specificity as the radiologists.

**Fig. 5:**
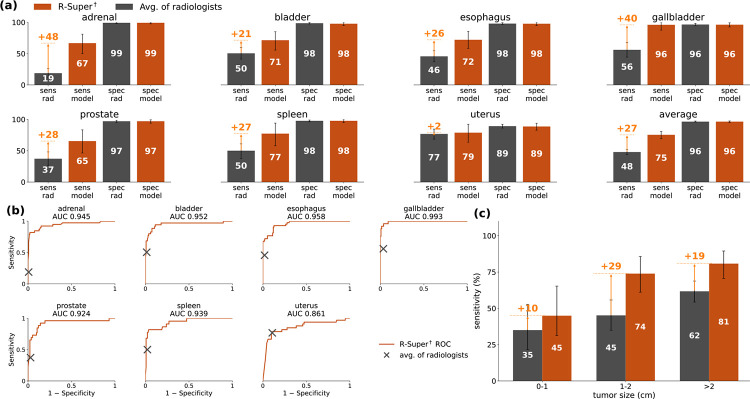
R-Super^†^ detected 56% more malignant tumors than the average radiologist (75% vs. 48% average sensitivity) at the same false-positive rate (96% specificity). Therefore, R -Super^†^ has potential to help radiologists detect tumors that would have been missed in clinical practice. **(a) R-Super**^**†**^ **surpassed the average sensitivity of 6 radiologists in malignant tumor detection for 6 tumor types, and matched the radiologists for uterus tumors**. R-Super^†^ was trained with reports and masks, and evaluated at approximately the same specificity as the radiologist average, on the same reader study dataset (Tab. 1). **(b) ROC curves for R-Super are above the radiologist average for 6 tumor types in malignant tumor detection**. **(c) R-Super**^**†**^ **surpasses radiologists in detecting small malignant tumors**. The bar plot shows sensitivity for R-Super^†^ and the radiologists, for different ranges of tumor diameters. R-Super^†^ surpassed the radiologists for detecting small tumors, especially in the range of 1 to 2 cm. Again, R-Super^†^ was evaluated at approximately the same specificity as the radiologists, the specificity values shown in (a). Error bars represent 95% confidence intervals, calculated with bootstrapping (1,000 bootstrap samples).

Figure 5 compares the average performance of all radiologists to R-Super^†^, trained with reports and masks, for malignant tumor detection (malignant vs no-tumor). Evaluated at approximately the same specificity as the radiologists (96%, probable level), R-Super^†^ achieved 75% sensitivity (69–81%) versus 48% (44–52%) for the radiologists, a +27% absolute improvement or +56% relative improvement in sensitivity (p<0.05). R-Super’s sensitivity was higher for all seven tumor types and significantly higher (p<0.05) for six (all except uterus, Tab. D42). Figure 5 also shows tumor detection sensitivity by tumor size. The largest gains of R-Super over the radiologist performance occurred for small tumors between 1 and 2 cm in diameter (+29% sensitivity), suggesting that R-Super has potential to assist radiologists in early cancer detection.

#### R-Super^†^ also improved tumor detection and malignancy identification.

In tumor detection, R-Super’s sensitivity surpassed the radiologists (p<0.05) for 6 tumor types (all except uterus) at matched specificity (Tab. D41). On average, R-Super^†^ surpassed radiologists by +22% absolute sensitivity at matched specificity (p<0.05), reaching 61% average sensitivity (57–65%) at 96% specificity (95–98%). In malignancy identification (malignant vs benign + no-tumor), R-Super^†^ surpassed the average radiologist sensitivity for 5 tumor types (p<0.05 for 3), at matched specificity (Tab. D43). The radiologists did not significantly surpass R-Super^†^ for any tumor type (Tab. D43). On average, R-Super^†^ reached 53% sensitivity (46–59%) at 95% specificity in malignancy identification, significantly surpassing radiologists by +14% sensitivity at matched specificity. Tables detailing all reader study results are in Supp. D.8.

#### Even without tumor masks, R-Super often matched or exceeded radiologist performance.

R-Super trained with reports alone surpassed the radiologist sensitivity for 5 tumor types in tumor detection (significant for 2), at matched specificity (Tab. D44). It significantly surpassed the radiologists on average, reaching 46% sensitivity (43–50%) vs 39% radiologist sensitivity (37–41%), at 96% specificity (95–98%). In malignant tumor detection, report-only R-Super surpassed the radiologists’ sensitivity in 4 tumor types (significant for 2, at matched specificity) and performed similarly on average (+5% sensitivity, but p>0.05; Tab. D45). In malignancy identification, report-only R-Super surpassed radiologists’ sensitivity for 4 tumor types (p<0.05 for 2), and performed similarly on average (+3% sensitivity at matched specificity, but p>0.05; Tab. D46).

Overall, this reader study demonstrated that R-Super and R-Super^†^ can match or surpass radiologists in a dataset focusing on small tumors. Therefore, the R-Super framework has the potential to assist radiologists detect early tumors that would have been missed.

## 3 Discussion

This study shows that routine radiology and pathology reports can serve as direct supervision for tumor segmentation. We introduce R-Super, a training framework that converts report-derived tumor descriptions into localized training signals, enabling AI models to learn tumor segmentation without tumor masks. The core advance is to use reports not merely as image-level labels or auxiliary text, but as descriptions of what the segmentation should contain: the number, size, volume, attenuation, rough anatomical location, and malignancy status of tumors. Because reports are already produced at scale in routine care, whereas tumor masks are scarce, expensive, and absent for many tumor types, R-Super changes the scaling logic of tumor segmentation. It enables AI models to learn segmentation from hundreds of thousands of CT-Report pairs, reduces dependence on masks, and provides a scalable path toward more comprehensive, interpretable, and accurate multi-tumor detection.

R-Super is the first public AI model shown to match or surpass radiologists in detecting 7 tumor types on CT: adrenal glands, bladder, esophagus, gallbladder, prostate, spleen, and uterus tumors (Fig. 2). Trained on 127,496 3D CT scans (42 million slices), R-Super substantially outperformed 9 prior public AI models, including public segmentation models (FLARE and ULS) and leading VLMs from Google, Microsoft, Stanford, and Alibaba (Fig. 2). Usually, reports are used to train VLMs, which can analyze medical images and write AI-made reports. However, these VLM-written reports missed most tumors in our test datasets (Fig. 2), as in previous studies [10, 39]. VLMs performed better in zero-shot classification than in report generation, but R-Super still surpassed them by large margins (Fig. 2). This could be attributed to a design difference. Current VLM architectures and training methods, such as CLIP [40], were originally designed for natural images and captions, which are often vague image descriptions. Medical reports contain rich tumor descriptions, including tumor measurements and approximate locations. The R-Super loss functions convert these descriptions into localized supervision for segmentation. This localized supervision helps R-Super detect tumors that can occupy less than 0.0001% of a CT.

R-Super’s performance scales with the number of training reports. We did not observe scaling saturation despite training with over 100,000 reports, representing 27 years of multi-tumor CT scans collected at UCSF and affiliated institutions (Fig. 3a). However, this scaling benefit heavily depends on the AI framework. A classifier trained on the same reports as R-Super, using tumor presence/absence labels extracted from the reports, performed substantially worse than R-Super (Fig. 4). These results show that large-scale report datasets require training frameworks that effectively exploit the detailed information contained in reports. Additionally, this large-scale training was computationally efficient: we trained R-Super in 10 days on one NVIDIA H100 GPU. R-Super also works with any segmentation architecture and adds no inference-time cost.

R-Super can exploit reports and masks together. Thirty-one radiologists created 870 tumor masks for our UCSF Train dataset, with the support of R-Super (Sec. C.2). Training with these CT-Mask pairs plus the CT-Report pairs substantially outperforms training with the CT-Mask pairs alone (Sec. 2.2). R-Super also surpasses previous methods that learned from reports and masks. CLIP [30] pre-trains AI models to pair CT scans and reports. Afterwards, the AI model is fine-tuned for tumor segmentation with CT-Mask pairs. Multi-task learning (MTL, [35]) trains AI models for classification using report-based labels, while masks train the model for segmentation. However, both MTL and CLIP use reports to train the AI for auxiliary tasks, not to directly supervise tumor segmentation. In contrast, R-Super’s loss functions directly supervise segmentation, by comparing segmented tumors to tumor descriptions in reports and penalizing discrepancies. This direct supervision allows R-Super^†^ to significantly surpass the alternative methods trained on the same dataset (Fig. 4).

R-Super could help radiologists detect cancer, according to our reader study with 6 radiologists. R-Super^†^ surpassed radiologists in malignant tumor detection for 6 tumor types and matched them for uterus tumors (Sec. 2.4). On average, R-Super^†^ detected 56% more malignant tumors than radiologists at matched specificity, on a dataset focused on small tumors (Fig. 5). These results suggest that R-Super could help radiologists detect small tumors that would otherwise be missed. The tumor types we studied are notably difficult to detect on CT: their signs are subtle, often imperceptible to the human eye. AI can perceive these signs, as previous studies demonstrated for individual tumor types [7], and our study demonstrates for multi-tumor detection.

R-Super performs in a clinically relevant range relative to established cancer-detection exams, though direct comparison is limited and should be read as context rather than equivalence. For spleen, gallbladder, and adrenal tumors, detection already depends largely on incidental findings in imaging, making AI-assisted detection on routine CT a natural objective that could potentially extend to other modalities such as MRI. For the remaining tumor types, specialized cancer-detection exams exist: cystoscopy and cytology for bladder, transvaginal ultrasound (TVS) for uterus, prostate-specific antigen (PSA) and digital rectal examination (DRE) for prostate, and endoscopy for esophagus. For prostate cancer, PSA achieves 21%/94% sensitivity/specificity (4.1 ng/mL cutpoint) [41], while DRE achieves 51%/59% [42], both below the performance of R-Super in our CT test sets (65%/96% sensitivity/specificity in prostatic malignancy identification, Tab. D43). For bladder cancer, urine cytology achieves 10 to 51% sensitivity and 83 to 88% specificity [43], also considerably below R-Super (57% sensitivity and 98% specificity, Tab. D43). However, this comparison is inherently limited: the reference cancer-detection exams were evaluated in populations different from our test datasets, and sensitivity and specificity are not directly transportable. Cystoscopy, endoscopy, and TVS surpass both R-Super and radiologists (cystoscopy: 97% sensitivity, 96% specificity [44]; endoscopy: 94% sensitivity, 92% specificity [45]; TVS: 81% sensitivity, 86% specificity [46]). However, we do not suggest that CT should replace dedicated cancer-detection exams. CT is performed at very large scale for many indications, registering unsuspected tumors that often remain undetected [5]. In contrast, high-accuracy cancer-detection exams are often used in smaller scale and prompted by symptoms or other risk factors. We therefore envision R-Super as an assistive tool that opportunistically flags suspicious findings for clinician review, who, when appropriate, can prompt targeted follow-up with established cancer-detection exams or interval imaging.

Whether this opportunistic use produces net clinical benefit will depend on model calibration. The R-Super performance in Fig. 3d corresponds to false-positive rates of 8 to 12%, which would likely create a substantial downstream workup burden (e.g., additional imaging, endoscopy, and short-interval follow-up) if applied to the large, low-cancer-prevalence population undergoing routine CT. Adrenal, prostate, and splenic lesions are recognized sources of overdiagnosis, and detecting additional small or indolent lesions may not improve outcomes and could lead to overtreatment. Therefore, clinical deployment would need low false-positive rates. In our reader study, R-Super operated at 99% specificity while still significantly surpassing radiologists by +25% sensitivity in malignant tumor detection (Tab. D50), suggesting that false-positive burden may be reduced substantially while preserving clinically meaningful sensitivity gains. However, future prospective evaluation must measure false-positive burden, downstream resource use, target population (general vs high-risk patients), and the proportion of flagged tumors that are clinically actionable. Additionally, R-Super is best positioned as a flagging system for clinician adjudication rather than autonomous diagnosis.

This study has limitations. First, tumor segmentation DSC was lower than is commonly reported for tumor types where CT is the primary detection modality (Supp. D.2). This likely reflects the intrinsic difficulty of outlining our studied tumor types on CT: many are small, subtle, poorly marginated, or poorly visible in CT. The same difficulty appears between humans: when different radiologists annotate these tumors, the DSC between their annotations is also relatively low (Supp. D.2). Nonetheless, R-Super^†^ surpassed the standard mask-based segmentation model by +14% DSC and accurately detected and localized tumors (Fig. 3b), even when contours were imperfect. This aligns with the primary goal of this study: accurate and interpretable tumor detection through segmentation, rather than radiotherapy-grade tumor contouring. Second, this study was retrospective. Although R-Super was evaluated internally and externally across multiple institutions in three continents, prospective multi-center studies are required before clinical implementation. Thus, the present study establishes technical feasibility, external generalization, and reader-study performance, but not yet prospective clinical utility. Third, R-Super uses an LLM to extract tumor information from reports during training. The LLM is highly accurate (up to 98% accuracy, Supp. D.4) but not perfect; yet R-Super achieves high tumor detection and segmentation performance and surpasses alternative training methods despite occasional LLM errors. Fourth, in our reader study, radiologists did not have access to the patients’ clinical history because this history often documented known cancers. This setup also simulates incidental detection, where patients often do not present with tumor-related symptoms or history. Previous reader studies on incidental detection used a similar no-history setup [7]. Finally, R-Super’s loss functions use organ segmentation masks during training, currently obtained from a public organ segmentation model [10]. Such public models cover dozens of organs [10, 29, 47], but not every organ, and their masks are also not perfect. R-Super has mechanisms to resist moderate organ mask errors (Sec. 4), and it substantially outperforms alternative training methods despite these errors (Fig. 4).

In conclusion, the R-Super results show that routine medical reports can become a scalable open-source foundation for multi-cancer detection and segmentation. Segmentation provides an accurate, interpretable, and verifiable framework for cancer detection, but it has been constrained by the need for voxel-wise tumor masks, which are expensive to create and unavailable for many tumor types. R-Super addresses this constraint by converting medical reports into localized training signals, allowing segmentation models to learn from CT-Report datasets orders of magnitude larger than CT-Mask datasets. Large-scale report supervision surpassed smaller-scale mask supervision for tumor detection and further improved performance when reports and masks were combined (Fig. 3a). Therefore, reports can substitute or supplement masks. By releasing the R-Super framework, trained models, CT-Report pairs, and new tumor masks, this study provides a public foundation for broader multi-cancer detection and segmentation. More broadly, it suggests that cancer imaging may undergo the same transition that has transformed other areas of AI: from small, manually annotated datasets toward large-scale learning from text that is readily available.

## 4 Methods

R-Super is a training framework designed to learn tumor segmentation from radiology and pathology reports. Unlike tumor masks, reports do not provide voxel-wise tumor locations. However, reports are available at large scale and provide detailed tumor descriptions: tumor count, diameters, rough locations (organ/slice), attenuation, and malignancy. Turning reports into effective and direct supervision for training tumor segmentation models required a new training framework (Fig. 1): first, R-Super extracts the tumor descriptions from each report using an LLM (Sec. 4.2); then, R-Super trains a tumor segmentation model with four new loss functions, which enforce that segmented tumors match these tumor descriptions extracted from reports:

*Volume Loss* ($L_{\text{vol}}$, Sec. 4.3): minimizes the dissimilarity between the tumor volume estimated from the segmentation output, and the tumor volume estimated from the radiology report.

*Ball Loss* ($L_{\text{ball}}$, Sec. 4.4): enforces segmented tumors to match the radiology report descriptions of tumor count, sizes, and rough locations. Given these report descriptions and the output of the segmentation model, the Ball Loss estimates the most probable per-voxel location of each reported tumor, and then maximizes tumor probabilities at those per-voxel locations. The paradigm of estimating a missing variable (here, per-voxel tumor locations) from an observable variable (the report) and a model, and using this estimate to update the model, is theoretically grounded on the Expectation-Maximization framework [48] (Sec. 4.7).

*Attenuation Loss* ($L_{\text{att}}$, Sec. 4.5): enforces segmented tumors to match the tumor attenuation in radiology reports (i.e., if tumors appear dark or bright).

*Malignancy Loss* ($L_{\text{mal}}$, Sec. 4.6): enforces segmented tumors to match the malignancy (cancer or not) described in pathology reports, when available.

The four losses combine into the R-Super Loss, $L_{\text{rsuper}}$, show in Eq. 1 (using weights $\lambda_{\text{vol}},\lambda_{\text{ball}},\lambda_{\text{att}},\lambda_{\text{mal}}$, set to 0.1, 0.1, 0.01, and 0.1 in all experiments). In the equation, x is the 3D CT scan, r the report, and $f\left(x;\theta_{s}\right)$ the output of the tumor segmentation model. Together, these losses exploit detailed report information but tolerate reports with missing details. Reports are used only during training, never at inference. If CT-Mask pairs are also available for training, we use standard segmentation losses (Dice and cross-entropy, Eq. 2) on CT-Mask pairs $\left(x,y\sim {𝒟}_{\text{mask}}\right)$, and the R-Super loss on CT-Report pairs $\left(x,r\sim {𝒟}_{\text{report}}\right)$.

For tumor detection, the segmentation output, $f\left(x;\theta_{s}\right)$, is processed by a classifier, $c\left(f\left(x;\theta_{s}\right);\theta_{c}\right)$, which outputs per-organ probabilities of tumor, benign tumor, and malignant tumor. The classifier is trained with cross-entropy loss $\left(L_{\text{cls}}\right)$ on classification labels (l) extracted from reports (Supp. C.1). Performing tumor detection based on segmentation improves accuracy and explainability (Fig. 3). The R-Super full training objective is shown in Eq. 3. Data pre-processing and training details are presented in Supp. E.


(1) $$L_{\text{rsuper}}\left(f\left(x;\theta_{s}\right),r\right)=\lambda_{\text{vol}}L_{\text{vol}}\left(f\left(x;\theta_{s}\right),r\right)+\lambda_{\text{ball}}L_{\text{ball}}\left(f\left(x;\theta_{s}\right),r\right)+\lambda_{\text{att}}L_{\text{att}}\left(f\left(x;\theta_{s}\right),r\right)+\lambda_{\text{mal}}L_{\text{mal}}\left(f\left(x;\theta_{s}\right),r\right)$$

(2) $$L_{\text{seg}}\left(f\left(x;\theta_{s}\right),y\right)=\text{Dice}\left(f\left(x;\theta_{s}\right),y\right)+\text{CE}\left(f\left(x;\theta_{s}\right),y\right)$$

(3) $$\underset{\theta_{s},\theta_{\text{c}}}{\text{min}}\left\{E_{(x,y)\sim {𝒟}_{\text{mask}}}\left[L_{\text{seg}}\left(f\left(x;\theta_{s}\right),y\right)\right]+E_{(x,r)\sim {𝒟}_{\text{report}}}\left[L_{\text{rsuper}}\left(f\left(x;\theta_{s}\right),r\right)\right]+E_{(x,l)\sim 𝒟}\left[L_{\text{cls}}\left(c\left(f\left(x;\theta_{s}\right);\theta_{c}\right),l\right)\right]\right\}$$

The R-Super loss functions leverage organ masks to locate the organs with reported tumors. To create these organ masks, we used an organ segmentation model, an nnU-Net [37] trained in AbdomenAtlas [10] (made public). Organ masks are pre-saved before training the tumor segmentation model. Although public segmentation models can segment few tumor types, they can segment dozens of organs accurately [29, 47]. To compensate for occasional errors in organ masks and account for tumors that grow beyond organs, we dilate these masks by 2 cm. R-Super trains any segmentation architecture, here, we used MedFormer [28], a U-Net-based CNN-transformer hybrid that won previous benchmarks [29].

### 4.1 A large-scale CT-Report Dataset

We assembled two training and five test datasets, allowing external validation in 3 countries (USA, Turkey, and Switzerland). Datasets are summarized in Tables 1 and A1. Together, the datasets include 134,476 different 3D CT scans and radiology reports, over 5 times the largest public CT datasets [21, 22]. Data was sourced from the PACS system of multiple hospitals: UCSF and affiliated institutions in California, USA; Medipol University Hospital, Turkey; and University Hospital Basel, Switzerland. Additionally, 25,494 CT scans were sourced from the public Merlin dataset (Stanford hospital) [22].

The datasets cover tumor types with no or minimal public CT masks: esophagus, bladder, gallbladder, spleen, uterus, prostate, and adrenal tumors. Only 53 public masks exist for adrenal tumors [19] (adrenocortical carcinoma only), 17 for esophagus tumors, and 39 for uterus tumors [20]. No public CT masks exist for the remaining tumor types. For each tumor type, our training dataset has from 2,527 to 14,050 CT-Report pairs. All data were de-identified prior to this study, and include chest, abdomen, and pelvic CT. Each patient may have multiple time points and contrast phases (Tab. 1).

Each dataset contains no-tumor cases, benign tumors, and malignant tumors (counts in Tab. A1). Here, benign tumors are generically defined as non-malignant focal lesions, encompassing cysts and solid lesions, identified by explicit benign characterization in radiology reports and absence of malignancy (in the benign tumor’s organ) across all available ICD-10 codes, radiology reports, and pathology reports. Malignant tumors include primary cancers and metastases (Tab. A1). In UCSF Test, malignancy was confirmed using pathology reports. In the remaining test datasets, where pathology reports were unavailable, malignancy was confirmed by explicit malignancy characterization in radiology reports. In the reader study dataset, malignancy was determined from pathology reports (UCSF data, 50%), or by explicit mentions of already diagnosed active cancer in radiology reports or clinical history (e.g., “Findings consistent with known cervical carcinoma”, or ongoing cancer treatment). Test control patients (no-tumor patients) had no mention of a current or prior tumor in any radiology/pathology report or diagnostic code, for all 7 organs we focus on. Control patients may nonetheless present with other abnormalities such as infection or aneurysm, which is important for realistic evaluation: R-Super must distinguish tumors from other pathologies, not only from healthy tissue. The training dataset contains pathology reports for part of the data (Tab. A1).

We will publicly release over 22,000 CT-Report pairs, and the first public tumor segmentation masks for multiple tumor types (Tab. 1). These masks were created by 31 radiologists assisted by R-Super, in an active learning loop (Sec. C.2). More details about each dataset, data acquisition, and train-test splits are in Supp. A.

### 4.2 LLM for Extracting Report Information

We use an LLM to extract tumor characteristics from reports. From radiology reports, it extracts tumor count, rough locations (organ/tumor slice), attenuation, and diameters. From pathology reports, it extracts whether tumors are malignant or benign. For training cases without pathology reports, the LLM looks for high-confidence confirmations of malignancy in the radiology report instead.

LLMs can extract information from reports created by different radiologists, in different styles, since LLMs can understand language semantics and context [10]. To keep the LLM applicable to different report styles and avoid overfitting to the reports in our training dataset, we use a zero-shot LLM, Qwen 3 30B (A3B Thinking FP8) [49]. To reduce computational cost, we run the LLM only once per report and store its answer. Our LLM prompt (Supp. G) was iteratively developed by radiologists and computer scientists through testing and error analysis. The prompt provides the LLM with medical knowledge and clear guidelines for interpreting reports. It also asks the LLM to justify its answers and fill a template with tumor characteristics. The template is automatically converted into a table, used as ground-truth for the Volume, Ball, Attenuation, and Malignancy Losses.

The LLM achieved 98.3% (95% CI: 97.6–98.9) accuracy and 95.8% F1-Score (94–97.4%) in determining whether tumors exist in each organ (i.e., determining tumor existence and rough location), 94.1% (91.2–96.7%) accuracy in determining malignancy, 95.6% (93.1–98.0) in determining size, and 92.9% (86.2–98.6%) in determining attenuation. The LLM evaluation is in Supp. D.4. This LLM performance surpasses methods used to extract information from reports and create ground-truth labels in popular medical datasets (e.g., 90% F1-Score in ChestX-ray8 [50]). Nevertheless, the LLM is not perfect, and occasional LLM errors exist in the R-Super training process. Despite this, R-Super substantially outperformed other training methods and public AI models (Fig. 4,2), indicating robustness to moderate LLM errors.

### 4.3 Volume Loss

Radiology reports do not inform the exact tumor contour, needed for usual segmentation loss functions. However, from reports we can estimate tumor volumes. The Volume Loss teaches the segmentation model to segment tumors that match tumor volumes estimated from the report. Specifically, it enforces two constraints: segmented tumors must be in the rough locations (organs) described in the report; and, for each *rough location* (o), the *combined volume* of all segmented tumors $\left(V_{s,o}\right)$ must match the combined volume of all tumors in the report $\left(V_{r,o}\right)$. We estimate the segmented tumor volume $\left(V_{s,o}\right)$ by summing per-voxel tumor probabilities (output of the segmentation model) within the rough location o (organ). We estimate the reported tumor volume $\left(V_{r,o}\right)$ from the tumor diameters described in the radiology report. The Volume Loss is designed to be easy to optimize and converge, as it only constrains overall tumor volumes, not tumor diameters or counts. We apply it to an intermediate layer of the segmentation model (e.g., the second decoder layer in MedFormer) to produce a rough segmentation. The remaining losses, applied to the last layer, then teach the segmentation model to refine this rough segmentation to match multiple tumor characteristics in the report. The Volume Loss is shown in Fig. 1.

#### Overall equation $\left(L_{\text{vol},\text{o}}\right)$.

Equation 4 summarizes the Volume Loss for an organ o. When tumors are reported in the organ, the term $L_{\text{forg},o}\left(V_{s,o},V_{r,o}\right)$ minimizes the dissimilarity between the segmented tumor volume $V_{s,o}$ and the reported tumor volume $V_{r,o}$ in the organ o. Meanwhile, the term $L_{\text{bkg},o}\left(T^{o}\right)$ penalizes tumor segmentations outside the organ o (e.g., spleen tumors segmented outside the spleen). $T^{o}$ is the segmentation model output channel for tumors in the organ o. For an organ (o) where the report mentions no tumor, we simply penalize the entire tumor segmentation output ($T^{o}$) using per-voxel cross-entropy with a zero target (Eq. 4). The overall volume loss ($L_{\text{vol}}$, Eq. 5) is the sum of the per-organ volume loss $L_{\text{vol},o}$ for all organs of interest (o∈𝒪). Next, we explain the calculation of each term in Eq. 4.


(4) $$L_{\text{vol},o}=\left\{\begin{array}{ll} L_{\text{forg},o}\left(V_{s,o},V_{r,o}\right)+L_{\text{bkg},o}\left(T^{o}\right), & \text{if tumors are reported in}\;o\\ \text{CE}\left(T^{o},0\right), & \text{otherwise} \end{array}\right.$$

(5) $$L_{\text{vol}}=\sum_{o\in 𝒪}L_{\text{vol},o}$$

#### Estimating tumor volumes from reports $\left(V_{r,o}\right)$.

Most reports do not include tumor volumes. They usually include tumor diameters, from which we estimate volumes. For each tumor, 1, 2 or 3 perpendicular diameters are provided. From the diameters, we estimate tumor volumes. With 1 tumor diameter $\left(d_{1}\right)$, we estimate tumor volume as the volume of a ball $\left(d_{1}^{3}\pi /6\right)$; with 3 diameters $\left(d_{1},d_{2},d_{3}\right)$, we use the volume of an ellipsoid $d_{1}d_{2}d_{3}\pi /6$; with 2 diameters $\left(d_{1}\right.\;\text{and}\;\left.d_{2}\right)$, we estimate the third as $d_{3}=\left(d_{1}+d_{2}\right)/2$, and use the ellipsoid volume. For each organ o, we estimate the volume of all tumors in the organ and sum them, estimating the reported tumor volume $V_{r,o}$.

#### Estimating tumor volumes from segmentation $\left(V_{s,o}\right)$ and background penalization $\left(L_{\text{bkg},o}\right)$.

We estimate $V_{s,o}$ from the segmentation model’s output $T^{o}=\left[t_{h,w,l}^{o}\right]$, the per-voxel tumor probabilities for tumors in organ o. Inside $T^{o}$, we first locate the organ o, using an organ segmentation mask ($O=\left[o_{h,w,l}\right]$; organ masks are AI-made, pre-saved, and dilated to compensate for errors and tumors growing beyond organs). Outside the organ, the background penalization term $\left(L_{\text{bkg},o}\right)$ minimizes the tumor probabilities estimated by the AI, using cross-entropy with target of 0 (Eq. 6, where ⊙ stands for element-wise multiplication). To estimate $V_{s,o}$, we sum all per-voxel tumor probabilities inside the organ, and multiply them by the voxel volume v (Eq. 7). If tumor probabilities were binary (0/1), Eq. 7 would be equivalent to counting the tumor voxels inside the organ. In reality, tumor probabilities $t_{h,w,l}^{o}$ are continuous, so $V_{s,o}$ is a soft estimation of tumor volume.


(6) $$L_{\text{bkg},o}\left(T^{o}\right)=\text{CE}\left((1-O)⊙T^{o},0\right)$$

(7) $$V_{s,o}=v\sum_{h,w,l}^{H,W,L}t_{h,w,l}^{o}o_{h,w,l}$$

#### Volume dissimilarity function and tolerating report errors $\left(L_{\text{forg},o}\right)$.

The Volume Loss minimizes the dissimilarity between the segmented tumor volume $\left(V_{s,o}\right)$ and the reported tumor volume $\left(V_{r,o}\right)$, by minimizing the dissimilarity function $L_{\text{forg},o}\left(V_{s,o},V_{r,o}\right)$ in Eq. 8. In the equation, E is a constant (set to 500 mm^3^) that provides numerical stability when $V_{r,o}$ is small. This function, plotted in Fig. 1, surpassed standard loss functions (e.g., L1 and L2 losses) in preliminary tests, and it has 3 design principles. First, it measures a dissimilarity between $V_{s,o}$ and $V_{r,o}$ : its minimum is when $V_{s,o}$ matches $V_{r,o}$, and it increases with the difference between $V_{s,o}$ and $V_{r,o}$. Second, it has a strong (but finite) gradient when $V_{s,o}$ is zero but $V_{r,o}$ is not. Therefore, the Volume Loss strongly penalizes the segmentation model for missing tumors mentioned in the report (false negatives). To further prevent false negatives, gradients are softer when $V_{s,o}$ is larger than $V_{r,o}$. Third, $L_{\text{forg},o}\left(V_{s,o},V_{r,o}\right)$ has a tolerance margin (created by the max operation in Eq. 8): if the difference between the segmented tumor volume and the reported tumor volume is small (we use a tolerance, τ, of 10%), $L_{\text{forg},o}\left(V_{s,o},V_{r,o}\right)$ and its gradient become zero, not penalizing the AI. We use this tolerance because $V_{r,o}$ is not a perfect measurement of the true tumor volume. Our estimation of $V_{r,o}$ from tumor diameters in the report is an approximation. Furthermore, the diameters in the report are subject to inter-observer variance (two radiologists measuring the same tumor often provide slightly different diameters). The Volume Loss tolerance (τ) compensates for these inaccuracies.


(8) $$L_{\text{forg},o}\left(V_{s,o},V_{r,o}\right)=\text{max}\left\{L_{\text{forg},o}^{\prime }\left(V_{s,o},V_{r,o}\right)-L_{\text{forg},o}^{\prime }\left((1-\tau )V_{r,o},V_{r,o}\right),0\right\}$$

(9) $$L_{\text{forg},o}^{\prime }\left(V_{s,o},V_{r,o}\right)=\frac{\left|V_{s,o}-V_{r,o}\right|}{V_{s,o}+V_{r,o}+E}$$

#### Exploiting tumor slices and reports missing tumor sizes or counts.

Reports may also provide the tumor slice, i.e., the vertical coordinate $z_{i}$ of a tumor i in organ o. When slices are provided, the Volume Loss becomes more precise, teaching the AI to only segment tumors near these slices. To this end, before applying the Volume Loss, we edit the organ mask $\left(\tilde{O}\leftarrow O\right)$ by setting it to zero at vertical coordinates more than one tumor diameter $\left(d_{i}\right)$ away from every reported slice $z_{i}$ (Eq. 10, where $𝒵=\left\{z_{i}\right\}$ is the set of slices for all tumors in organ o, and x,y,z are spatial coordinates). This restriction makes the segmented tumor volume $V_{s,o}$ depend only on regions near the reported slices (Eq. 7), encouraging tumor segmentation there. Meanwhile, the $L_{\text{bkg},o}$ term of the Volume Loss (Eq. 6) penalizes segmentations far from the slices. The Volume Loss can also handle reports missing tumor sizes or tumor counts (e.g., reports that only measure and count the largest tumors). In these cases, we employ a prior-based, high-tolerance reformulation of the Volume Loss, explained in Supp. C.3.


(10) $$\tilde{o}(x,y,z)=\left\{\begin{array}{ll} 0, & \text{if}\;\left|z-z_{i}\right|>d_{i}\;\forall z_{i}\in 𝒵\\ o(x,y,z), & \text{otherwise} \end{array}\right.$$

#### Cropping requirement.

Medical segmentation models are typically trained on CT crops rather than whole scans. The Volume Loss needs the crop to fully contain the organ with tumors. Otherwise, the Volume Loss could encourage the model to segment a tumor that is not inside the crop. We randomly choose on which organ to crop on, giving higher priority (90%) to organs with tumors. If a second organ with tumor is partially inside the crop, its tumor channels receive no loss.

### 4.4 Ball Loss

Radiology reports inform not only approximate tumor volumes, but also diverse tumor characteristics. The Ball Loss teaches the segmentation model to segment tumors that match the radiology report in tumor count, diameters, and rough locations (organs / slice). Thus, the Ball Loss is more strict than the Volume Loss, and it is applied to the last layer of the segmentation model. In summary, the Ball Loss uses the output of the tumor segmentation model and the tumor descriptions in radiology reports to locate the most probable position for each tumor described in the report. In each position, the Ball Loss optimizes the segmented tumor to match the corresponding tumor size (diameter and volume) from the report. As an example (Fig. 1), consider a report mentions a 30 mm spleen tumor. The Ball Loss first locates the spleen. Inside the spleen, it locates the 30 mm sphere where the tumor probability (predicted by the segmentation model) is highest. Inside this sphere, the Ball Loss maximizes the top-N most probable voxels, where N is the tumor volume estimated from the report (Sec. 4.3), in voxels, minus a tolerance margin (20%). Around these top-N most probable voxels, an uncertainty margin is not optimized, compensating for uncertainty at tumor borders. Outside this margin, tumor probabilities are minimized. Therefore, the Ball Loss enforces the segmentation of a single spleen tumor of any shape that fits inside a sphere of 30 mm, the tumor diameter in the report. The Ball Loss is summarized in Fig. 1.

#### Overall Equation $\left(L_{\text{ball},o}\right)$.

For organs o where the report mentions no tumor, the Ball Loss minimizes the AI tumor segmentation output $\left(T^{o}\right)$, by minimizing a cross-entropy with target 0 (Eq. 11). For organs with reported tumors, we define the Ball Loss as $L_{\text{ball},o}^{\prime }$, calculated with three operations: ball convolution, finding of the most likely tumor voxels, and maximization of tumor probabilities in these voxels. These operations are explained below. The final Ball Loss, aggregated over all organs of interest (o∈𝒪), is shown in Eq. 12.


(11) $$L_{\text{ball},o}=\left\{\begin{array}{ll} L_{\text{ball},o}^{\prime }, & \text{if tumors are reported in}\;o\\ \text{CE}\left(T^{o},0\right), & \text{otherwise} \end{array}\right.$$

(12) $$L_{\text{ball}}=\sum_{o\in 𝒪}L_{\text{ball},o}$$

#### Ball Convolution: locating the most likely tumor positions.

First, we use pre-saved organ masks **O** (AI-made organ masks dilated by 2 cm) to locate the organ o in the tumor segmentation output $T^{o}$ (per-voxel tumor probabilities). Inside the organ o, we locate each tumor described in the report. Initially, consider that organ o has a single reported tumor i. To locate it, we use an operation that we called *Ball Convolution*: a standard 3D convolution (stride 1 and zero-padding), but with a non-learnable kernel shaped like a ball ($k_{i}$, defined in Eq. 13). This ball is binary (1 inside, 0 outside) and has the same diameter $d_{i}$ as the tumor diameter in the report (the largest reported diameter for tumor i). We apply the Ball Convolution to the tumor segmentation output (tumor probabilities, $T^{o}$), multiplied by the organ mask (O), as shown in Eq. 14, where * stands for convolution. The Ball Convolution moves the ball (kernel) over the tumor segmentation output. At each position, the ball sums all the tumor probabilities inside it. Therefore, the maximum output of the Ball Convolution locates the most probable center (c) for a tumor with the reported diameter d (Eq. 15, where x,y,z are spatial coordinates). I.e., a ball of diameter d and center c represents the most probable per-voxel tumor location, the *highest probability ball* (H), defined in Eq. 16. In the equation, μ represents a small dilation (we set μ=20%), used to compensate for inaccuracies in the reported diameter d. The Ball Convolution cannot set the highest probability ball center c outside the organ o, due to the previous multiplication between the tumor segmentation output and the organ mask (Eq. 14).


(13) $$k_{i}(u,v,w)=\left\{\begin{array}{ll} 1, & \text{if}\;\sqrt{u^{2}+v^{2}+w^{2}}\leq \frac{d}{2}\\ 0, & \text{otherwise} \end{array}\right.$$

(14) $$B=\left(O⊙T^{o}\right)*k_{i}$$

(15) $$c=\left[c_{x},c_{y},c_{z}\right]=\underset{\left(x,y,z\right)}{\text{argmax}\;}B(x,y,z)$$

(16) $$H(x,y,z)=\left\{\begin{array}{ll} 1, & \text{if}\;\|(x,y,z)-c\|\leq \frac{d(1+\mu )}{2}\\ 0, & \text{otherwise} \end{array}\right.$$

#### Locating the most likely tumor voxels.

The Ball Loss teaches the AI to segment a tumor that fits inside the highest probability ball (H), but this tumor does not need to be spherical. To allow for various tumor shapes, we do not maximize all voxels inside the highest probability ball, but only its top-N voxels with the highest tumor probabilities. We define N as the tumor volume estimated from the report (as in Sec. 4.3), reduced by a small tolerance margin (20%), and converted to a voxel count (N). The top-N voxels can assume any shape that fits inside the highest probability ball, and they have a reasonable volume, considering the tumor volume in the report. The top-N voxels represent the most likely tumor voxels given the segmentation output and the tumor description in the radiology report (tumor organ and diameter).

#### Maximization and minimization of tumor probabilities.

The Ball Loss maximizes the tumor probabilities in these top-N most probable tumor voxels. To this end, we define a segmentation mask M, where the top-N voxels are set to 1, and other voxels to 0 (Eq. 17). We use this mask as the target for cross-entropy and Dice losses applied to the segmentation model’s output $T^{o}$. However, to account for the segmentation model’s uncertainty at tumor borders, we introduce an uncertainty margin consisting of the next most probable voxels within the highest probability ball, i.e., the top-[N+1,νN] voxels (we set ν=1.2). This margin is stored in an uncertainty mask $M^{\prime }$ (Eq. 18). We skip penalization of these uncertain voxels by masking the segmentation model’s output $T^{o}$ with $\left(1-M^{\prime }\right)$ before the CE and Dice losses, preventing gradient propagation and optimization in this uncertain region. The Ball Loss with this uncertainty margin is defined in Eq. 19. The masks M and $M^{\prime }$ are generated continuously in training, improving as the segmentation model improves.


(17) $$M(x,y,z)=\left\{\begin{array}{ll} 1, & \text{if}\;(x,y,z)\in {\text{top}}_{\left[1,N\right]}\left(T^{o}(x,y,z)⊙H(x,y,z)\right)\\ 0, & \text{otherwise} \end{array}\right.$$

(18) $$M^{\prime }(x,y,z)=\left\{\begin{array}{ll} 1, & \text{if}\;(x,y,z)\in {\text{top}}_{\left[N+1,\nu N\right]}\left(T^{o}(x,y,z)⊙H(x,y,z)\right)\\ 0, & \text{otherwise} \end{array}\right.$$

(19) $$L_{\text{ball},o}^{\prime }=\text{CE}\left(\left(1-M^{\prime }\right)⊙T^{o},M\right)+\text{Dice}\left(\left(1-M^{\prime }\right)⊙T^{o},M\right)$$

#### Reports with multiple tumors.

When reports mention multiple tumors in an organ o, we locate them from largest to smallest. For each tumor i, we repeat the previously explained procedure: a Ball Convolution identifies the tumor’s highest-probability ball, the top-N most probable voxels inside this ball are added to the mask $M_{i}$, and the top-[N+1,νN] voxels are added to the uncertainty mask ${M^{\prime }}_{i}$. After each step, these selected top-N voxels are removed from the tumor segmentation output $\left(T^{o}\right)$ so they cannot be reused for the next tumor. After we locate all tumors reported in organ o, we compute the union of their individual masks, generating the joint masks M and $M^{\prime }$ (Eq. 20). These joint masks become the target for the cross-entropy and Dice losses in Eq. 19.


(20) $$M=\underset{i}{⋃}M_{i},\;M^{\prime }=\underset{i}{⋃}M_{i}^{\prime }$$

#### Exploiting tumor slices and reports missing tumor sizes or counts.

The Ball Loss leverages the available information reports, becoming more precise when reports provide more tumor details. When reports include tumor slices, the Ball Loss teaches the AI to segment tumors at the reported slices. To this end, we adapt each Ball Convolution to locate a tumor near a reported slice, by temporarily zeroing the organ mask in slices more than 1 tumor diameter away from the reported tumor slice (Eq. 10, but setting 𝒵 to a single slice $z_{i}$ for each Ball Convolution, $𝒵=\left[z_{i}\right]$). This makes the Ball Convolution search region very small, making the Ball Loss very precise. The Ball Loss is also usable for less precise reports that lack the number or diameters of tumors. In these cases, we use a high-tolerance reformulation of the Ball Loss, which teaches the segmentation model to segment at least the number of tumors explicitly reported in the radiology report (see Supp. Note C.4).

### 4.5 Attenuation Loss

The Attenuation Loss constrains tumor segmentation to be consistent with the tumor attenuation described in radiology reports (Fig. 1). Attenuation encodes the relative brightness of the tumor with respect to the surrounding organ (hypo-, hyper-, or isoattenuating). To calculate the Attenuation Loss, we first estimate brightness statistics. Specifically, the mean and standard deviation of the organ voxels and the tumor voxels in the CT ($\mu^{o},\sigma^{o},\mu^{t,o}$, and $\sigma^{t,o}$, respectively). These four statistics are given to a small attenuation classifier (MLP with 128 hidden neurons). It classifies whether the tumors are hypoattenuating, hyperattenuating, or mixed/isoattenuating. The label for the attenuation classifier is extracted from the report by the LLM. We train the classifier with a standard cross-entropy classification loss. The gradient of this loss trains the attenuation classifier, but it also back-propagates to the segmentation model, because the statistics $\mu^{t,o}$ and $\sigma^{t,o}$ depend on the tumor voxels, which are defined according to the segmentation model’s output $T^{o}$ (Eq. 23 to 25). The gradient encourages the segmentation model to help the attenuation classifier: it should segment tumors whose attenuation match the report, so that the attenuation classifier can correctly classify their attenuation. The Attenuation Loss is applied both as deep supervision and at the last layer of the segmentation model.

#### Formulation.

The Attenuation Loss for organ o is shown in Eq. 21, where the attenuation classifier is represented as function $a\left(\mu^{o},\sigma^{o},\mu^{t,o},\sigma^{t,o};\theta_{a}\right)$, with parameters $\theta_{a}$. In case there is no tumor reported in the organ o or when reports do not inform attenuation, the loss is set to 0; otherwise, it its set as the attenuation classifier cross-entropy loss. The attenuation label, extracted from report, is encoded as an integer A∈0,1,2 (representing hypo-, hyper-, and iso-/mixed attenuation, where mixed indicates that different tumors in organ o have different attenuation). Eq. 22 shows the loss aggregated for all organs of interest (o∈𝒪). Organ and tumor brightness statistics $\left(\mu^{o},\sigma^{o},\mu^{t,o},\sigma^{t,o}\right)$ are defined in Eq. 23 and 24. These statistics are calculated from the CT scan X (normalized per-voxel HU values), the segmentation model’s output $T^{o}$ (tumor probabilities), and the pre-saved organ segmentation mask O.


(21) $$L_{att,o}=\left\{\begin{array}{ll} \text{CE}\left(a\left(\mu^{o},\sigma^{o},\mu^{t,o},\sigma^{t,o};\theta_{a}\right),A\right), & \text{if tumors are reported in}\;o\\ 0, & \text{otherwise} \end{array}\right.$$

(22) $$L_{att}=\sum_{o\in 𝒪}L_{att,o}$$

(23) $$\mu^{o}=\frac{\sum_{h,w,l}m_{h,w,l}^{o}x_{h,w,l}}{\sum_{h,w,l}m_{h,w,l}^{o}+\varepsilon },\;\sigma^{o}=\sqrt{\frac{\sum_{h,w,l}m_{h,w,l}^{o}{\left(x_{h,w,l}-\mu^{o}\right)}^{2}}{\sum_{h,w,l}m_{h,w,l}^{o}+\varepsilon }}$$

(24) $$\mu^{t,o}=\frac{\sum_{h,w,l}t_{h,w,l}^{o}x_{h,w,l}}{\sum_{h,w,l}t_{h,w,l}^{o}+\varepsilon },\;\sigma^{t,o}=\sqrt{\frac{\sum_{h,w,l}t_{h,w,l}^{o}{\left(x_{h,w,l}-\mu^{t,o}\right)}^{2}}{\sum_{h,w,l}t_{h,w,l}^{o}+\varepsilon }}$$

(25) $$m_{h,w,l}^{o}=\left(1-t_{h,w,l}^{o}\right)o_{h,w,l}$$

### 4.6 Malignancy Loss

The Malignancy Loss $\left(L_{\text{mal}}\right)$ trains the segmentation model to identify tumors as benign or malignant. From pathology reports, the LLM extracts whether each tumor is malignant (cancer) or benign. Thus, by using the LLM to analyze pathology and radiology reports together, we define the diameter, rough location (organ / slice), and malignancy of each tumor. For CT scans with tumors of inconclusive malignancy (e.g., a benign and a malignant tumor in the same organ with similar sizes or an inconclusive report), we skip the Malignancy Loss. For scans without pathology reports, we extract malignancy from the radiology report instead, if it includes malignancy information (e.g., radiology reports often mention the patient has a known active cancer). The segmentation model includes separate output channels for segmenting general tumors, malignant tumors, and benign tumors (3 channels per organ). The Volume, Ball, and Attenuation Losses train the tumor channels, while the benign and malignant channels are trained exclusively by the Malignancy Loss.

#### Formulation.

The Malignancy Loss is a variation of the Ball Loss (Sec. 4.4), beginning with the same procedure: Ball Convolutions are applied to general tumor segmentation output of the segmentation model ($T^{o}$) to identify, for each reported tumor, the most probable tumor voxels given the reported tumor size and rough location; these voxels define the tumor mask $M_{i}$, while the surrounding voxels (uncertain tumor borders) define the uncertainty mask ${M^{\prime }}_{i}$ (Eq. 13 to 18). Afterwards, unlike the Ball Loss, the Malignancy Loss merges the masks for benign and malignant tumor separately, producing the $M^{b}$ and $M^{\prime b}$ joint masks for benign tumors, and $M^{m}$ and $M^{\prime m}$ for malignant tumors, as defined in Eq. 26. In the equation, ${ℬ}^{o}$ and ${ℳ}^{o}$ denote the sets of benign and malignant tumors in organ o, respectively, as determined from reports. The joint masks for benign and malignant tumors ($M^{b}$ and $M^{m}$) are used as targets for Dice and CE losses, which supervise the segmentation model’s outputs for benign and malignant tumors ($T^{o,b}$ and $T^{o,m}$), as shown in Eq. 27 and 28. As in the Ball Loss, uncertainty masks ($M^{\prime b}$ and $M^{\prime m}$) define voxels that are not optimized. If organ o contains no benign / malignant tumor (and no tumor of unknown malignancy), the corresponding benign / malignant segmentation output is minimized with a cross-entropy loss with target zero (Eq. 29 and 30). The final Malignancy Loss over all organs of interest (o∈𝒪) is given in Eq. 32. The benign and malignant tumor masks $\left(M^{m},M^{b}\right)$ that guide the Malignancy Loss are derived from the same general tumor output $T^{o}$ (and reports), enforcing consistency in the segmentation model outputs for benign, malignant, and overall tumors.


(26) $$M^{b}=\underset{i\in {ℬ}^{o}}{⋃}M_{i},{\;M}^{\prime b}=\underset{i\in {ℬ}^{o}}{⋃}M_{i}^{\prime },{\;M}^{m}=\underset{i\in {ℳ}^{o}}{⋃}M_{i},{\;M}^{\prime m}=\underset{i\in {ℳ}^{o}}{⋃}M_{i}^{\prime }$$

(27) $${L^{\prime }}_{\text{mal},o}^{b}=\text{CE}\left(\left(1-{M^{\prime }}^{b}\right)⊙T^{o,b},M^{b}\right)+\text{Dice}\left(\left(1-{M^{\prime }}^{b}\right)⊙T^{o,b},M^{b}\right)$$

(28) $${L^{\prime }}_{\text{mal},o}^{m}=\text{CE}\left(\left(1-{M^{\prime }}^{m}\right)⊙T^{o,m},M^{m}\right)+\text{Dice}\left(\left(1-{M^{\prime }}^{m}\right)⊙T^{o,m},M^{m}\right)$$

(29) $$L_{\text{mal},o}^{b}=\{\begin{array}{ll} {L^{\prime }}_{\text{mal},o}^{b}, & \text{if benign tumors are reported in}\;o\\ \text{CE}\left(T^{o,b},0\right), & \text{otherwise} \end{array}$$

(30) $$L_{\text{mal},o}^{m}=\{\begin{array}{ll} {L^{\prime }}_{\text{mal,}o}^{m}, & \text{if malignant tumors are reported in}\;o\\ \text{CE}\left(T^{o,m},0\right), & \text{otherwise} \end{array}$$

(31) $$L_{\text{mal,}o}=L_{\text{mal,}o}^{b}+L_{\text{mal,}o}^{m}$$

(32) $$L_{\text{mal}}=\sum_{o\in 𝒪}L_{\text{mal,}o}$$

### 4.7 Rationale and Robustness of the Report Supervision Losses

#### Does the Ball Loss work if the segmentation model cannot find the tumor?

The Ball Loss remains effective even when the AI output is weak (i.e., all predicted per-voxel tumor probabilities are low). In this case, the Ball Convolutions still find the most probable tumor voxels, and maximize probabilities in these voxels. If the AI model predicted only zero probabilities, the Ball Convolution would fail. However, this situation is unlikely, because the Volume Loss strongly penalizes the AI when all predicted tumor probabilities are zero but the report mentions tumors. The Volume Loss supports the Ball Loss.

#### Can the Ball Loss reinforce false positives?

In a single training image, if the model segments a false positive inside an organ that contains a reported tumor (i.e., a tumor segmented in the wrong part of the organ), the Ball Loss may temporarily reinforce that false positive. However, this mistake will not be reinforced consistently across the training dataset. When a similar false positive appears in a no-tumor patient, the Ball Loss will penalize it. To minimize the Ball Loss across the whole training dataset, the segmentation model must learn to segment tumors that match multiple tumor characteristics in reports: tumor rough locations, counts, and sizes. The Attenuation and Malignancy Losses add further constraints by requiring segmented tumors to match the reported tumor attenuation and malignancy. False positives do not match all of these characteristics consistently across patients, but true tumors do. In general, loss functions are known to potentially enforce incorrect solutions for individual training samples (e.g., false positive tumors), but minimizing these losses over the full dataset favors solutions that reduce error consistently across samples [51, 52] (e.g., correct tumor segmentations). Consistent with this, our new report-based losses substantially improved tumor detection and localization compared with training on CT-Mask pairs alone (Sec. 2.2). This result indicates that, across the full training dataset, the new losses guide the model toward true tumors rather than false positives.

#### Relationship to theory on learning from incomplete data.

Theoretically, the Ball Loss is inspired by the principles of the Expectation–Maximization (EM) algorithm, a classical statistical framework for learning from incomplete data problems [53]. In such problems, part of the data cannot be directly observed (here, the per-voxel tumor segmentation mask), and another part can be observed (here, report descriptions of tumor counts, diameters, and rough locations). At a high level, EM alternates between estimating the unobserved data from the observed data and the current model (Expectation step), and updating the model so that its outputs better match the estimated unobserved data. In an adaptation to object segmentation in photographs [54], the Expectation step consisted in estimating the most likely object mask (unobserved data) given a segmentation model’s output and image-level binary classification labels (observed data); the Maximization step then optimized the segmentation model to better match this most likely object mask. The Ball Loss follows an EM-like structure: it presents a new algorithm (based on the Ball Convolution) that estimates a most likely tumor mask given the output of a segmentation model and the detailed tumor description in the report; then, it optimizes the segmentation model to better match this most likely tumor mask. As in classical EM, repeating this procedure leads to a progressive improvement of both the model and the estimates of the unobserved data: as the segmentation model improves, the estimated most likely tumor masks improve, resulting in increasingly accurate supervision.

The Volume Loss does not follow an EM-like formulation, as it does not estimate a most likely tumor mask. Instead, it directly calculates the tumor volume from the segmentation model’s output and enforces this volume to match the tumor volume estimated from the report. The accuracy of EM-based models is known to depend on initialization [55]. At a high level, applying the Volume Loss to hidden layers of the segmentation model is akin to an EM initialization procedure: it encourages the segmentation model to produce a reasonable coarse tumor mask, which later layers can refine according to the Ball Loss. Our ablation studies show that the Volume Loss is very important for accuracy (Supp. D.5), as initializations are important for EM.

#### Statistical Analysis.

We calculated 95% confidence intervals with bootstrapping with 1,000 samples with replacement. For significance testing we used paired permutation tests, with 10,000 permutations, except for comparisons of AUC. For AUC, we used DeLong’s test. All reported P values are two-sided.

## Supplementary Material

Supplementary Files

This is a list of supplementary files associated with this preprint. Click to download.

- nrreportingsummaryrsuperfilled.pdf
- Supplementary.pdf

## Figures and Tables

**Table 1:** Our multi-tumor CT datasets. We created two training datasets and four test datasets to train and evaluate R-Super. More than 22,000 CT-Report pairs will be publicly released. We directly collected data from hospital PACS systems in three countries: UCSF (USA), Medipol (Turkey), and Basel (Switzerland). Stanford data (25,494 scans) were obtained from the public Merlin dataset [22]. For internal validation on UCSF and external validation on Medipol and Basel, models were trained on R-Super train, which combines UCSF and Stanford data. For external validation on Stanford, models were trained on UCSF train, which excludes Stanford data. The reader study dataset includes cases from UCSF (internal validation), Medipol (external validation), and Stanford (external validation).

	R-Super train	UCSF train	UCSF test	Stanford test	Medipol test	Basel test	reader study
CT scans	127,496	116,514	2,301	1,976	1,327	2,935	637
patients	58,258	43,164	939	1,689	577	2,229	513
controls	13,584	10,614	113	114	178	54	132
**source**							
country	USA	USA	USA	USA	Turkey	Switzerland	USA & Turkey
hospitals	UCSF & affiliates (80.3%) Stanford (19.7%)	UCSF & affiliates	UCSF & affiliates	Stanford	Medipol	Basel	UCSF & affiliates (50.1%) Stanford (30.1%) Medipol (19.8%)
**age and sex**							
mean age (SD)	57.7 (18.0)	59.4 (17.0)	62.2 (15.7)	61.9 (16.7)	53.8 (15.4)	68.5 (13.2)	61.5 (15.7)
female, no. (%)	31,557 (54.2)	22,856 (53.0)	445 (47.4)	996 (59.0)	259 (44.9)	1,017 (45.6)	260 (50.8)
male, no. (%)	26,701 (45.8)	20,308 (47.0)	494 (52.6)	693 (41.0)	318 (55.1)	1,212 (54.4)	253 (49.2)
**spacing**							
in-plane, mm	0.82 (0.47)	0.84 (0.50)	0.83 (0.46)	0.76 (0.09)	0.82 (0.09)	0.79 (0.25)	0.80 (0.28)
slice thickness, mm	2.00 (1.57)	2.23 (1.69)	2.25 (1.90)	1.14 (0.18)	1.43 (0.48)	1.05 (0.30)	1.75 (1.23)
**contrast phase**							
venous, no. (%)	91,956 (72.1)	69,925 (60.0)	1,494 (64.9)	1,941 (98.2)	1,128 (85.0)	920 (31.3)	516 (81.0)
arterial, no. (%)	8,691 (6.8)	8,827 (7.6)	187 (8.1)	19 (1.0)	136 (10.2)	192 (6.5)	30 (4.7)
non-contrast, no. (%)	12,833 (10.1)	13,553 (11.6)	0 (0.0)	2 (0.1)	0 (0.0)	39 (1.3)	0 (0.0)
unknown, no. (%)	14,016 (11.0)	24,209 (20.8)	620 (26.9)	14 (0.7)	63 (4.7)	1,784 (60.8)	91 (14.3)
**tumors**							
bladder	4,065	4,050	345	209	146	295	86
gallbladder	3,780	3,562	265	200	174	76	65
prostate	2,527	2,458	180	105	35	170	72
uterus	6,516	5,792	302	330	132	269	104
esophagus	2,720	3,141	388	40	189	95	78
spleen	14,050	13,223	474	494	357	691	95
adrenal gland	11,955	11,477	667	801	357	1,417	127
**masks**							
total	1,420	870	–	602	–	–	–
bladder	180	88	–	95	–	–	–
gallbladder	141	97	–	76	–	–	–
prostate	156	90	–	68	–	–	–
uterus	288	206	–	91	–	–	–
esophagus	241	191	–	51	–	–	–
spleen	238	142	–	101	–	–	–
adrenal gland	176	56	–	120	–	–	–

## Data Availability

The R-Super custom code is publicly available at GitHub (https://github.com/PedroRASB/SegmentationFromReports). The implementation builds on open-source libraries, including Python (v.3.10) and PyTorch (v.2.10).

## Data Availability.

This paper is based on large-scale CT datasets, including 127,496 CT scans in R-Super train, 116,514 in UCSF Train, 2,301 in UCSF Test, 1,976 in Stanford Test, 1,327 in Medipol Test, 2,935 in Basel Test, and 637 in the reader study dataset. Each CT scan is paired with its corresponding radiology report. The Stanford data (25,494 CT scans and reports) come from the public Merlin dataset, available at https://stanfordaimi.azurewebsites.net/datasets/60b9c7ff-877b-48ce-96c3-0194c8205c40. The remaining data was acquired by our team from hospital PACS, converted from DICOM to NifTI, and de-identified by removing all patient-identifiable metadata. We already have authorization to release 22,000 CT scans and the corresponding reports. Authorization to release the remaining data is pending. Besides CT scans and reports, tumor masks created by 31 radiologists will also be made publicly available. The de-identified data will be made available upon publication through HuggingFace under a CC-BY-NC-ND-4.0 license.
